# Retraction Note: PAK4, a target of miR-9-5p, promotes cell proliferation and inhibits apoptosis in colorectal cancer

**DOI:** 10.1186/s11658-025-00730-w

**Published:** 2025-04-16

**Authors:** Meihua Wang, Qianqian Gao, Yufang Chen, Ziyan Li, Lingping Yue, Yun Cao

**Affiliations:** Department of Pathology, Changzhou Tumor Hospital Affiliated to Soochow University, 68 Honghe Road Changzhou, Changzhou, 213032 Jiangsu China

**Retraction Note: Cellular & Molecular Biology Letters (2019) 24:58** 10.1186/s11658-019-0182-9

The Editor-in-Chief has retracted this article because it contains data that overlaps with data from the following article, [1], also published by *Cellular & Molecular Biology Letters*. In addition, an investigation conducted after the publication of the two articles, which were under consideration by the journal at the same time, found additional signs which raise concerns about the authorship of the two manuscripts and the circumstances of the research presented in them. The Editor-in-Chief therefore no longer has confidence in the results and conclusions presented in this article.

The authors have not replied to correspondence from the Publisher.
